# Assessment the performance of chemical constituents of agro wastes in production safety alternative carbon black filler in rubber composite purpose

**DOI:** 10.1038/s41598-025-92404-y

**Published:** 2025-04-01

**Authors:** Vivian F. Lotfy, Altaf H. Basta, Emad S. Shafik

**Affiliations:** 1 Cellulose and Paper Department, National Research Centre, Dokki, Giza 12622 Egypt; 2 Polymers and Pigmemts Department, National Research Centre, Dokki, Giza 12622 Egypt

**Keywords:** Agro-waste, EPDM rubber, Carbon black, Safety co-filler, Renewable, Rubber composite, Environmental sciences, Natural hazards, Chemistry, Materials science

## Abstract

Recently, minimizing petroleum resources as well as safely disposing of agro-wastes are essential for the production process to comply with environmental legislation. Bio-filler as an alternative to non-safety carbon black (CB) from petroleum resources in the production of rubber composites is investigated by many researchers, but unfortunately it leads to deterioration of the properties of rubber composites. To avoid this drawback, different agro-wastes (rice straw, date palm fiber, and reed (*Arundo* *donax* L.) with different chemical constituents as precursors of biofillers (biochars) are assessed toward the performance of ethylene-propylene-diene terpolymer rubber (EPDM). The role of replacing parts of CB with biochar on the rheological characteristics, physico-mechanical properties, hardness, swelling, and crosslinking density of EPDM composites is studied. The results proved the efficient low replacing ratio of biochar towards increasing the minimum and maximum torque; this indicates a homogeneous filler structure and crosslinking interactions between the components matrix as emphasized from the morphological analysis of EPDM rubber. The reverse trend is noticed on increasing the replacement ratio over 25%, where it deteriorates the tensile strength in comparison to pristine CB. The data demonstrated the most efficient biochar, which is derived from RS. The formulation containing 75% CB and 25% RS-biochar provided EPDM with tensile strength (14.4 MPa), higher than the pure CB (12.45 MPa). Moreover, this optimum formulation provided high crosslinking density, high hardness shore A, and swelling resistance of motor oil and toluene when compared to EPDM with pure carbon black. This promising finding trend is not noticed in the literature on using biochars, which usually caused the deterioration in properties of rubber products.

## Introduction

In recent years, the efforts of international communities are concerned to decrease the consumption of non-renewable resources, particularly fossil fuels, given their severe environmental consequences on climate change and global warming. According to the International Energy Agency (IEA), the emission of carbon dioxide related to energy worldwide has increased by 1.1% in 2023, with coal accounting for more than 65% of emissions^1^. This can be a warning to both humanity and the environment. The focus on renewable resource-based materials, as green engineered materials, has increased due to growing environmental concerns, landfill limitations, and waste management^2–4^. Over the past two decades, there have been ongoing studies of polymer blends made from renewable resources, which involve combining components derived from biomass. Our previous efforts to valorize lignocellulosic materials as a renewable resource for the development of functional lignocellulose products, are focused on production hydrogel delivery^5,6^, artificial wood^7–9^, building materials^10,11^, advanced nanoarchitectonics^12^, rubber composites^13–16^ and materials for carbon nanostructures (ACs, and CNTs)^17–20^.

One of the most widely manufactured carbon nanostructured materials is carbon black (CB), of which 70% is utilized as a pigment and a reinforcing phase in rubber and plastics^21^. It is produced from the incomplete burning of petroleum products, or coal tar that causes many environmental hazards. Consequently, the production of carbon black (CB) in an environmentally friendly and sustainable manner was investigated by the utilization of new feedstock materials derived from renewable sources or the recycling of waste. Bio-based natural residues are gaining popularity as green alternatives and inexpensive fillers due to their biodegradability, help the environment protection and achieving the sustainable development standards. Moreover, they enhanced the performance of rubber products, and reduced costs^22^. Environmentally friendly and sustainable additives like biochar have become utilized by the rubber industry; it can be produced by subjecting agro-wastes to high temperature and pressure in a pyrolytic reactor in presence or absence of oxygen^23,24^. Biochar is a solid material with a low fiber structure and a high carbon content that is produced from a variety of agricultural, forestry, aquatic, and industrial residues. Hydrophobicity, long-term stability, chemical composition, high porosity, high surface area, and other characteristics are some of the distinctive properties of biochar^25,26^. The cost of producing biochar must be carefully compared to that of carbon black based on market pricing. In 2023, the cost of producing carbon black is estimated to be between $1198 and $2085 per metric ton. The average cost of biochar in the same year was $131 per metric ton.

Ethylene propylene diene monomer (EPDM) is a synthetic rubber that is widely used because it has a saturated backbone. This backbone provides exceptional thermal resistance and stability to oxygen and ozone. EPDM is frequently utilized as thermal insulation in products including jacketing, profiles, seals, and cable insulation for different industrial fields including automotive, construction, and aerospace industries. The unfilled EPDM rubbers have relatively weak mechanical properties, so incorporating reinforcing fillers is crucial to providing strength for the rubber^27^.

In this study, the effective role of biochar from different agro wastes [rice straw (RS), date palm fiber (DP), and reed (*Arundo* *donax* L.) giant reed (GR)] as biofiller to EPDM composites is assessed. The EPDM composites are filled by biochar in hybrid with carbon black (CB). The idea of this investigation is to study the synergistic role of biochar containing silica in various ratios with CB as safety reinforcing rubber fillers. The optimum formulation is estimated from the rheological and physic-mechanical characteristics of the final rubber vulcanizates.

## Materials and experimental techniques

### Materials

Ethylene propylene diene monomer rubber (EPDM–Buna EPT 9650) contained 53% of ethylene chains and 6.5% of ethylidenenorbornene (ENB) chains, with 63MU Mooney viscosity (ML 1 + 4, 125 °C). It is a product of LANXESS Buna, Germany. Chemicals of commercial-grade such as stearic acid and zinc oxide (ZnO) as Activators, paraffin oil as (Plasticizer), *N*-Cyclohexyl-2-benzothiazole sulphenamide (CBS), tetramethylthiuram disulfide (TMTD), as accelerators, and elemental sulfur (Curing agent) were used without any further purification. 2,2,4-trimethyl-1,2-dihydroquinoline (TMQ) as antioxidant is a commercial-grade product. High abrasion furnace (HAF) carbon black N330 (CB) was obtained from Amerya Company for carbon black, Egypt. The motor oil (Mobil Super 15W-50 XHP) is a product of Exxon Mobil.

### Experimental techniques

#### Preparation of biochar

Three types of agriculture wastes [rice straw (RS), date palm fiber (DP) and reed (*Arundo* *donax* L.) giant reed (GR)] are used for preparation of biochar using pyrolysis technique. These agricultural wastes are collected from Egyptian fields, washed, dried at 105 °C and subjected to grinding. The dried samples are pyrolyzed in a muffle furnace in absence of air for 1 h at 400 °C. The average yield of biochar from RS, DP and GR are 48.2, 60.8 and 46.7%, respectively.

#### Characterization of agro-waste fiber and biochar

- Proximate analysis and chemical constituents of agro-fibers are characterized according to the standard methods. The agriculture waste samples are weighed both before and after all of the organic matter was burned, heated to 450 °C for 1 h, then elevated to 800 °C in a muffle furnace and held there for 45 min to determine the ash content. Quantification analysis of the contents of lignin^28^, pentosans, and α-cellulose was also done using standard procedures of the institute of paper chemistry No. 428 (1951) and 424(1952)^29,30^.
- Particle size distribution of different biochar powders was calculated using the screening method across various size sieves ranging from 53 to 500 µm (Germany standard testing sieve; Retsch). A parallel study was carried out by using the Laica Stereo microscope, which showed the different particle sizes, and supported the screening method with macroscopic photos.
- X-ray fluorescence spectroscopy (XRF**)**: The chemical composition of the biochar powders was determined by XRF technique using Axios, Sequential mod. (WD-XRF), PANalytical 2005 (Netherlands).

#### Preparation, rheological characteristics and vulcanization of EPDM rubber composites

In accordance with ASTM D3182-21a^31^, ethylene propylene diene monomer rubber (EPDM) was combined with other compounding components and curatives and placed into an open two roll mill with a 170-mm diameter, a 300-mm working distance, and a 24-rpm slow roll speed at a 1:1.25 gear ratio. Based on parts per 100 parts of rubber, the basic recipe for EPDM rubber formulations in this study includes ZnO_4_ phr, stearic acid 2 phr, antioxidant (TMQ) 1 phr, paraffin oil 3 phr, TMTD 1 phr, CBS 0.8 phr, and sulfur 2.2 phr. For 2 min, EPDM was masticated onto the two-roll mill in the first step. After that, ZnO, stearic acid, and the antioxidant TMQ were added and combined for 2 min for every component. Subsequently, the EPDM rubber was mixed for 5 min with HAF330 and biochar fillers added. The accelerators (TMTD and CBS) were then added to the rubber mixtures along with sulfur, and each component was stirred for 2 min.

The rheological properties of the rubber blends were evaluated by using a moving dierheometer MDR-one rheometer (TA Instrument) at 152 ± 1 °C in accordance with ASTM D2084-19a^32^. Maximum torque (M_H_), minimum torque (M_L_), scorch time (ts_2_), optimal cure time (Tc_90_), and cure rate index (CRI) were measured. To compute the cure rate index by the following Eq. (1), the EPDM samples was subjected to vulcanization using a hydraulic press and a clean, polished mold (15 cm × 15 cm × 0.2 cm), at their ideal cure times (Tc_90_), pressure of 4 MPa and temperature 152 ± 1 °C.

1 $$Cure \; rate \; index \; \left(CRI\right)=\frac{100}{Tc90-ts2}$$

#### Physico-mechanical properties of EPDM vulcanizates

The samples preparation for these properties was achieved using an ASTM cutter to cut the, EPDM sheet vulcanizates into dumbbell-shaped specimens that were 5 cm long and 0.4 cm wide. A standard thickness gauge was used to measure the thickness of each specimen. The Zwick/Roell Z010 tensile tester machine with load cell (Type: X force P and Nominal force: 10 KN) was used to determine the physico-mechanical properties (tensile strength, MPa, elongation at break, %, and hardness shore A) of the rubber composite vulcanizates both before and after accelerated thermal aging in accordance with ASTM D412-16 (2021)^33^.

An average of five replicates of the physico-mechanical data was obtained. In accordance with ASTM D573-04 (2019)^34^, accelerated thermal aging was performed from 1 day to 7 days at 90 °C in an air-circulated oven. The following formulae (2, 3) are used to determine the maintained values of tensile strength (TS) and elongation at break (E %) during thermal aging:

2 $$\text{Retained} \; \text{Tensile} \; \text{strength},{\%}=\frac{Ts \; after \; aging}{Ts \; before \; aging}\times 100$$

3 $$\text{Retained} \; \text{Elongation} \; \text{at} \; \text{break}, {\%}=\frac{E \; after \; aging}{E \; before \; aging}\times 100$$

#### Swelling test and crosslinking density for EPDM vulcanizates

By calculating the weight swell at equilibrium (Q %) from the following Eq. (4), the swelling behavior of the rubber vulcanizates was determined in toluene and motor oil, according to ASTM D471-97^35^.

4 $${Q\%}=\frac{{W}_{s}-{W}_{d}}{{W}_{d}}\times 100$$

where W_s_ is the weight of the specimen after swelling, and W_d_ is the weight of the dry specimen. The average of five replicates was considered.

The crosslinking density of the rubber vulcanizates was computed from the results of the equilibrium swelling in toluene, using the Flory-Rehner Eq. (5)^36^.

5 $${M}_{c}= -\rho {V}_{s}{V}_{r}^\frac{1}{3} / \left[{\ln}\left(1-{V}_{r}\right)+{V}_{r}+X{V}_{r}^{2}\right]$$

where *ρ* is the density of rubbers, V_s_ is the molar volume of the solvent, *X* is the interaction parameter of rubbers where the *X* of (EPDM) is 0.49, V_r_ is the volume fraction of swollen rubber and can be obtained from the masses and densities of rubber sample and the solvent. The degree of crosslinking (ν) (mol/cc) is given by Eq. (6):

6 $$\upnu = \frac{1}{2{M}_{c}}$$

#### Tear strength (tear resistance)

Under the same testing conditions as tensile strength, the tear strength of certain selected specimens was measured in accordance with ASTM D 624^37^. The tear strength was determined using the following formula (7).

7 $$Tear \; strength \; \left( {KN/m} \right) = ~\frac{{Load \; failure}}{{Thickness}}$$

### Surface morphology analysis

The morphology of a few selected rubber vulcanizates (a blank sample with 100% carbon black, 25% and 100% RS-biochar substituted CB) was examined using a scanning electron microscope (SEM) using a Quanta FEG250 system that drove the electron beam at 20 kV accelerating voltage after the samples were coated with gold.

## Results and discussion

### Characterization of agro-waste fiber and biochar

The chemical constituents of three agro-waste fibers under investigation, namely: RS, DP, and GR, as potential biochar precursors, are displayed in Table 1. In comparison between them, it has been noted that the giant reed (*Arundo* *donax* L.) has higher holo-cellulose (e.g., α-cellulose and hemicellulose) content and a lower lignin and ash content. The reported values were 83.3% holo-cellulose, 11.7% lignin, and 2.6% ash. Conversely, the RS fiber had a greater lignin and ash percentage (20.4% and 15.8%, respectively) and lower holo-cellulose content (56.6%). Regarding date palm fibers (DP), the percentages of holo-cellulose, lignin, and ash showed moderate values (18.5%, 5.8%, and 69.9%, respectively). All the measured values agree with the previously published data^38–40^. The percentages of their extractives using methanol-benzene as organic solvents, that corresponded to low and medium polarity compounds, were 8.38% for RS, 15.7 for DP, and 2.36% for GR. One factor that may influence the production yield of biochar is the proportion of ash and extractives in the residue during pyrolysis. As a result, the low yield of GR biochar (46.7%) may be due to its low ash and extractive content (2.6 and 2.36%), whereas RS and DP (48.2% and 60.8%, yield correspondingly) exhibit the opposite phenomenon due to their high ash and extractive content (15.8 and 8.38% for RS) and (5.8 and 15.7% for DP).

**Table 1:** Proximate analysis and chemical constituents of lignocellulosic biomass fibers (biochar precursors).

Code	Extractive, %	Lignin, %	Holo-cellulose, %	α-cellulose, %	Hemi-cellulose, %	Ash, %
RS	8.38	20.44	56.64	43.61	13.02	15.89
DP	15.76	18.55	69.96	38.99	24.79	5.820
GR	2.36	11.76	83.33	67.74	15.59	2.604

**Fig. 1:**
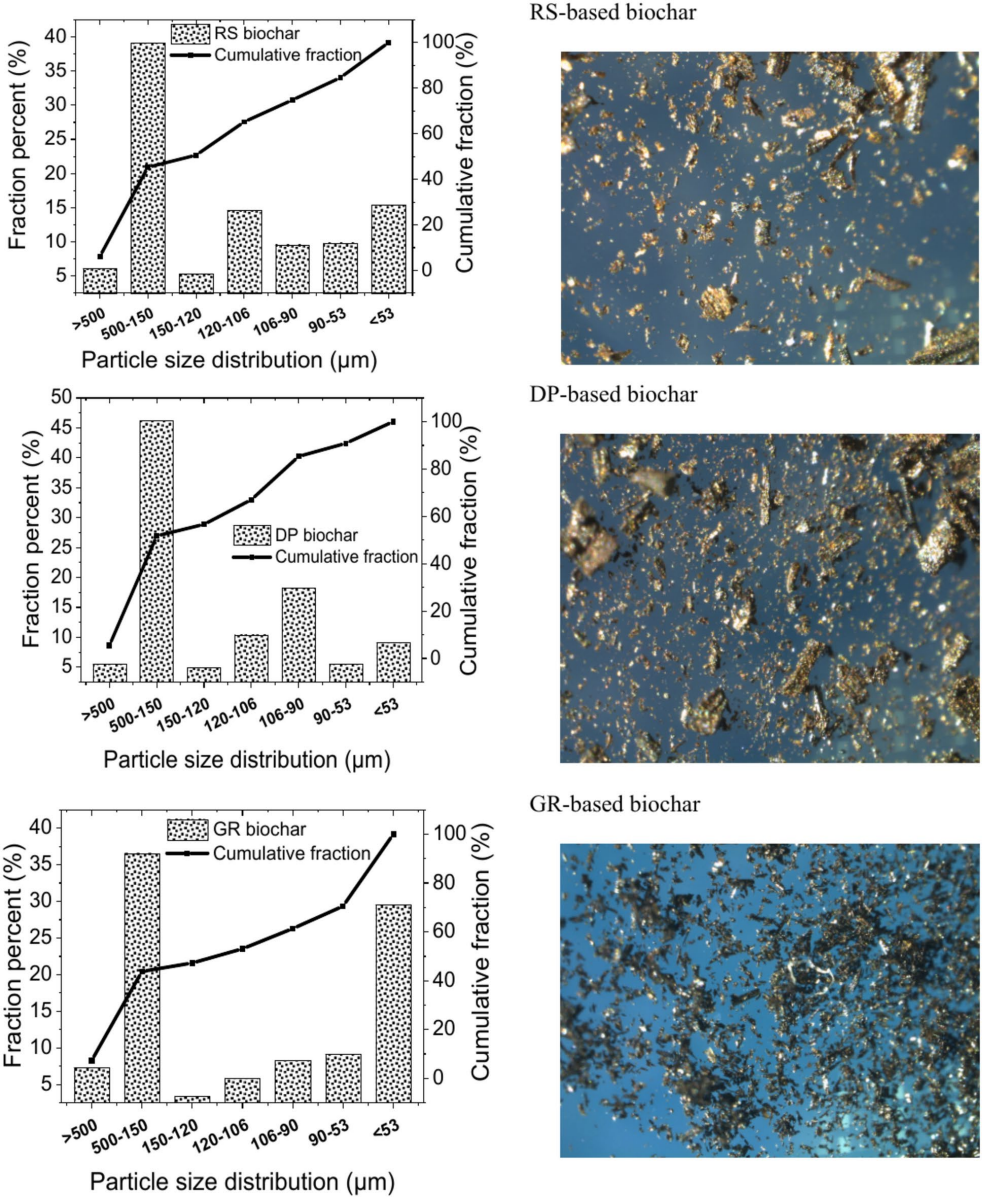
Sieved particle size distribution of investigated biochar in cumulative sum chart supported by macroscopic photos of biochar using the Laica stereo microscope.

Figure 1 displays the sieved particle size distribution of the investigated biochar in a cumulative sum chart enabling a qualitative examination of the size of the biochar. Three categories (fine, medium, and coarse) biochar can be used to group the measurements of biochar size. Of all the biochar studied, the fine biochar falls between 53 and 106 µm, consisting up around 33–47%. While the medium (106–500 µm) and coarse (more than 500 µm) and biochar’s represent 46–61% and 5–7%, respectively. This suggests that, with an average size of less than 500 µm, the examined biochar includes a majority of fine and medium-sized particles. The stereomicroscopic images support the emphasized view where the particle size of biochar is a mixture of fine and medium-sized particles. The biochar of RS and DP is mainly medium while the GR-biochar is fine.

Table 2 illustrates the chemical composition (e.g., major and minor inorganic components) of all biochar based on X-ray fluorescence (XRF) analysis. The major and minor inorganic components found in the pyrolyzed biochar were derived from the biomass of plants. When biochar is burnt at 400 °C, the main constituents present are SiO_2_, K_2_O, NaO, P_2_O_5_, Fe_2_O_3_, SO_3_, CaO, and MgO. The SiO_2_ content of all the biochar is found to be low to moderate; corresponding to 22.4, 3.68, and 1.99 weight percent for the RS, DP, and GR biochar, respectively. The composition of Fe_2_O_3_, P_2_O_5_, and SO_3_ is found to be comparable. Compared to other biochar, RS biochar has greater content of Si, Na, and K. In contrast, DP biochar has greater levels of Al, Ca, and Mg than other types. It has been observed in earlier research that at pyrolysis temperatures of 400–600 °C of biomasses, inorganic elements tend to remain in biochar after pyrolysis^41^.

**Table 2:** Chemical composition of the biochar produced from different precursors determined by XRF.

Main constituents	RS, (wt. %)	DP, (wt. %)	GR, (wt. %)
SiO_2_	22.40	3.680	1.990
TiO_2_	0.010	0.010	–
AL_2_O_3_	0.080	0.120	0.030
Fe_2_O_3tot_	0.100	0.120	0.150
MgO	0.710	0.890	0.240
CaO	0.670	1.150	0.170
Na_2_O	1.130	0.110	0.080
K_2_O	2.090	0.830	2.010
P_2_O_5_	0.240	0.180	0.220
SO_3_	0.230	0.370	0.230
Cl	0.670	0.170	0.290
LOI	71.60	92.27	94.54
MnO	0.065	0.043	0.008
CuO	0.003	0.004	0.009
ZnO	0.010	0.004	0.005
Co_3_O_4_	0.011	0.011	0.025
Br	0.010	0.018	0.004
Cr_2_O_3_	–	0.008	–
NiO	–	0.004	–
SrO	–	0.003	–

LOI implies loss on ignition.

### Rheological characteristics of EPDM rubber composites

The values of the minimum torque (M_L_), maximum torque (M_H_), optimum curing time (Tc_90_), scorch time (ts_2_), and cure rate index (CRI) are compiled in Table 3. In an unvulcanized elastomer, M_L_ represents the stiffness and viscosity value. M_H_ gauges the rigidity of compounds. The time it takes to attain 90% of the maximum torque is known as Tc_90_. On the other hand, ts_2_, which represents the time needed for the material to vulcanize, is the amount of time needed to increase the minimum torque by two units^42,43^. Furthermore, the rate of the vulcanization process is represented by the curing rate index (CRI).

**Table 3:** Rheometric properties of EPDM composites.

Sample	M_L_ (dN m)	M_H_ (dN m)	ΔM (dN m)	ts_2_ (min)	Tc_90_ (min)	CRI (min^-1^)
EPDM/100CB	3.14	36.45	33.31	1.48	10.46	11.13
EPDM/(75CB:25RS)	3.25	37.44	34.19	1.53	9.48	12.58
EPDM/(50CB:50RS)	3.09	35.62	32.53	2.06	10.01	12.58
EPDM/(25CB:75RS)	3.03	33.97	30.94	2.25	10.80	11.70
EPDM/(0CB:100RS)	3.00	30.31	27.31	3.44	12.05	11.61
EPDM/(75CB:25 DP)	3.25	36.71	33.46	1.49	10.11	11.60
EPDM/(50CB:50 DP)	3.65	37.16	33.51	1.59	10.13	11.71
EPDM/(25CB:75 DP)	3.78	39.23	35.45	2.30	10.43	12.30
EPDM/(0CB:100 DP)	3.35	32.92	29.57	3.42	12.28	11.28
EPDM/(75CB:25GR)	3.15	38.35	35.2	1.48	9.4	12.62
EPDM/(50CB:50GR)	3.39	37.79	34.4	1.48	10.54	11.04
EPDM/(25CB:75GR)	3.57	37.46	33.89	2.19	10.34	12.27
EPDM/(0CB:100GR)	3.47	35.03	31.56	3.39	11.58	12.21

M_L_, minimum torque; M_H_, maximum torque, ΔM, cure rate index, t_S2_, scorch time, tc_90_, optimum cure time, CRI, cure rate index.

There are variations in the viscosity and processability as shown by the M_L_ values for the EPDM composites filled with carbon black and varying ratios of biochar/carbon black. The M_L_ value of the majority of samples slightly increased when biochar was used in place of carbon black because it caused a little rise in viscosity. The high values of minimum torque (M_L_) indicate a more homogeneous filler structure, leading to more occlusion in the rubber matrix. In comparison to EPDM/100CB, most samples showed an increase in M_H_, which is a measure of the stiffness of elastomeric materials, with the substitution of biochar. Crosslinking interactions are suggested, which show great stability in the EPDM matrix and are responsible for the rise in M_H_.

For ts_2_, the low biochar substitution ratios (25 and 50%) demonstrated a similar or slightly increased scorch time 1.48–1.59 min (ts_2_ for EPDM/100CB 1.48 min). This suggests the EPDM rubber with CB substituted with 25 and 50% biochar requires the same amount of time to vulcanize. When carbon black in EPDM composites is replaced with 75 and 100% of biochar, undesirable outcomes are obtained; the scorching time increased to 2.19–3.44 min. While optimum vulcanization time (Tc_90_) values decreased for CB/biochar ratios (25–75%) and increased once more when carbon black was replaced completely with biochar. Lower values for the optimal vulcanization time (Tc_90_) enable the formulations to be produced in a shorter time, reducing energy and process costs. It was found that when the amount of biochar filler increased from 25 to 75% with respect to CB in the EPDM composites, the differential ∆M mostly increased. Therefore, the crosslinking density and efficiency of EPDM composites are improved by the incorporation of biochar^44^. The partial and complete replacement of CB with biochar increased the cure rate index (CRI) values, indicating that there was in fact a good interaction between the carbon black, biochar with the polymer matrix.

#### Effect of biochar on physic-mechanical of EPDM composites

**Fig. 2:**
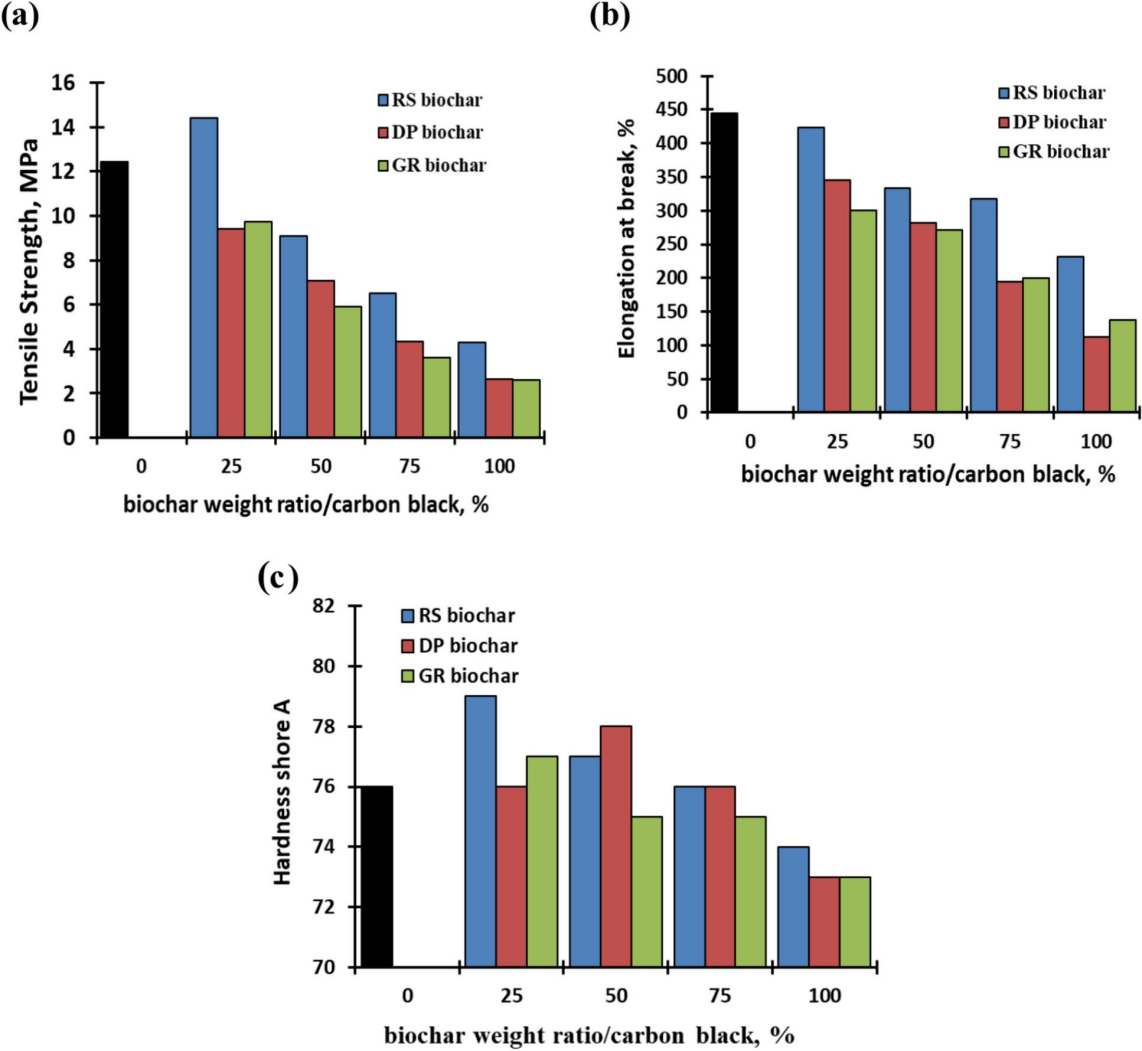
Mechanical properties of EPDM composites.

The variations in tensile strength, elongation at break, and hardness shore A for EPDM composites with 100% CB and various biochar replacement ratios are shown in Fig. 2a–c. As compared to pristine CB, the results clearly show that the tensile strength rapidly falls with increasing the biochar ratio, with the exception of EPDM containing75% of CB with 25% RS biochar (Fig. 2a). The tensile strength of EPDM composites with 100% carbon black was 12.45 MPa, but EPDM composites with 25% carbon black replaced with biochar from RS had 14.4 MPa. When the ratio of biochar with respect to CB increased, the tensile strength fell; for 100% biochar of RS, DP, and GR, the values were 4.3, 2.66, and 2.6 MPa, respectively. According to a prior study, poor tensile strength at high biochar loading was related to fewer biochar-polymer interfaces, which decreased polymer adhesion and increased agglomeration of biochar particles, resulting in lower mechanical properties^45,46^. Previous research reported the lower tensile strength values for rubber filled with biochar composites^47–49^. Comparing biochar resulted from giant reed and date palm to biochar made from rice straw; the results indicate a drop in tensile strength parameters. This can be attributed to the higher percentage of silica (26%) of RS-biochar than other biochar varieties (3.68 for DP-biochar and 1.99 for GR). According to research by Rashid et al., and Mokhothuet et al.^50,51^, tensile strength and elongation at break were enhanced by the addition of 10–20% weight percentage of silica nanoparticles.

Similarly, the addition of biochar is a negative influence on the elongation at break; the elongation is reduced from 444% for EPDM and 100% carbon to 232, 113, and 138 for 100% of RS, DP, and GR biochar (Fig. 2b). In comparison to other biochars, it was found that applying RS-biochar resulted in higher elongation values at break. This might be attributed to the high silica content of RS-biochar, where silica is considered one of the most widely used traditional reinforcement fillers^52^.

The impact of biochar type and filler loading on hardness is also depicted in Fig. 2c. When compared to the blank EPDM rubber that contained routine filler (100% CB), the figure illustrates the increase in hardness that occurs when RS-biochar is added. The hardness of blank sample was 76; it rose to 79 when 25% RS-biochar was substituted; however, the hardness of the EPDM composites containing 50, 75, and 100% RS-biochar declined as the biochar concentration grew to become 77, 76, and 74, respectively. The hardness value was comparable to blank rubber for other biochar substitutions at a 25% biochar substitution; however, it dropped to 73 for complete replacement.

The suggested mechanism of biochar preparation and its interaction with the EPDM matrix includes activation, physical adsorption and chemical bonding. Activation is the process of heating agricultural waste to high temperatures without the presence of oxygen during the pyrolysis process. As a result, biochar is produced, which has a surface rich in functional groups including hydroxyl, carboxyl, and phenolic groups and a highly porous structure. The EPDM matrix can interact with these groups. Dipole–dipole interactions, hydrogen bonds, or van der Waals forces are some of the ways that the functional groups on the surface of biochar might interact with the EPDM polymer chains via the physical adsorption. The chemical bonding includes covalent bonds that may occasionally occur between the surface groups of biochar and the polymer chains, particularly when using modified or active biochar. For example, biochar with oxygen-rich groups (such as hydroxyl or carboxyl) may undergo crosslinking processes that strengthen the bonding or react with the EPDM’s epoxide groups.

A comparative evaluation of the tensile strength and elongation at break of our investigated EPDM composites with literature dealing several rubbers with various fillers is listed in Table 4. According to Jiang et al.^53^, the mechanical properties of styrene butadiene rubber (SBR) were improved from 1.39 to 9.86 MPa by applying 40 phr of lignin biochar. The tensile strength dropped from 14.9 to 9.5 MPa when utilizing citrus tree trim-biochar with a variable ratio of biochar substituted for 100% carbon black^48^. The tensile destruction of natural rubber was reported to decrease from 18 to 7 MPa and from 25.1 to 17.8 MPa, respectively, when biochar derived from hard wood or paulownia was combined with calcium carbonate and soy protein^54,55^. The tensile strength of EPDM was slightly increased by 5.9% from 10.1 to 10.7 MPa by the stearic acid modified biochar made from citrus tree trim waste when 25% of the modified biochar was substituted for 100% CB^56^. Additionally, 10phr MDF biochar increased the tensile strength of EPDM somewhat, from 1.49 to 1.98 MPa^57^. In our investigation, we used RS-biochar, which increased the EPDM’s tensile strength from 12.45 to 14.4 MPa with an increment value of 15.66%.

**Table 4:** Comparative tensile strength and elongation at break of different rubbers with different fillers.

Matrix used	Filler	Filler content				Tensile strength (MPa)	Elongation at break, %	Reference
Styrene butadiene rubber (SBR)	Lignin biochar (LB)	0–40 phr	0			1.39	212	^53^
			40			9.86	507	
Styrene butadiene rubber (SBR)	Citrus tree trim-biochar	Substituted with CB (0, 25, 50, 75, 100%)	SBR/100CB			14.9	633	^48^
			SBR/25bio + 75CB			13.5	723	
			SBR/50bio + 50CB			11.2	709	
			SBR/75bio + 25CB			9.5	815	
			SBR/100biochar			6.97	775	
Natural rubber (NR)	Hard wood- biochar	0–50 phr biochar				Decreased from 18 to 7	Decreased from 450 to 280	^54^
		0–50 phr CB				Increased from 18 to 25	(470–350)	
Natural rubber (NR)	Paulownia -biochar	100% CB (43phr)				25.1	451	^55^
		100% biochar				17.8	547	
		40–50% of the CB replaced calcium carbonate, soy protein, and biochar				19.8–24.9	535–621	
Ethylene propylene diene monomer (EPDM)	Citrus tree trim-biochar modified with stearic acid	Substituted with CB (0, 25, 50, 75, 100%)			EPDM	6.22	495	^56^
					EPDM/40phr CB	10.1	570	
					EPDM/40phr biochar	8.77	600	
					EPDM/25biochar + 75CB	10.7	576	
Ethylene propylene diene monomer (EPDM)	MDF-biochar	EPDM				1.49	186	^57^
		EPDM/ 10 phr CB				3.02	244	
		EPDM/10 phr Biochar				1.98	199	
Ethylene propylene diene monomer (EPDM)	Rice-straw biochar	Substituted with CB (0, 25, 50, 75, 100%)		EPDM				Present study
				EPDM/40phr CB		12.45	444	
				EPDM/40phr biochar		4.30	232	
				EPDM/25biochar + 75CB		14.4	424	

#### Swelling test and crosslinking density for EPDM vulcanizates

**Fig. 3:**
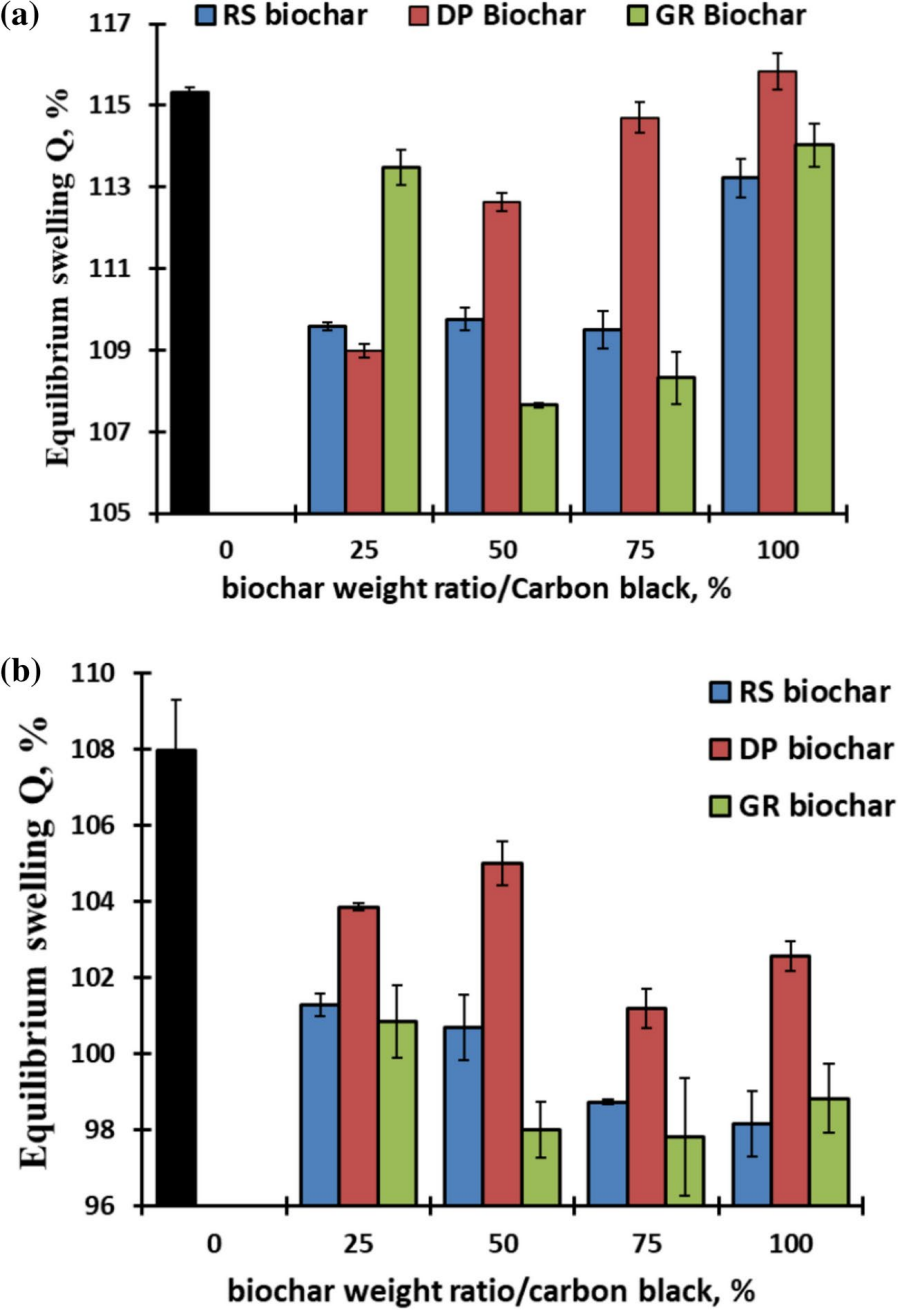
Equilibrium swelling (Q %) of EPDM composite in (**a**) toluene and (**b**) in motor oil versus carbon black and biochar weight ratios.

According to Mayasari and Yuniari^58^, swelling occurs as a result of the polymer expanding due to the availability of more free volume that assist in the mass transfer of the solvent. It is used to evaluate the interaction between the filler and the rubber matrix. Figure 3a,b illustrates the impact of various biochar ratios on the swelling ratio in toluene and motor oil of EPDM composites. As the CB substituted various types of biochar, the swelling ratio dropped in comparison to the neat CB-EPDM findings. Additionally, the findings demonstrated that, when compared to the same percentages of DP and GR biochar, RS-biochar is the most recommended because of its low equilibrium swelling in toluene (Fig. 3a). Swelling values dropped from 115.3 (EPDM with 100% CB) to around 109 (EPDM with 25–75% RS biochar) and 113.2% (EPDM with 100% RS biochar). The swelling results correlate with the higher mechanical properties of the EPDM rubber containing RS-biochar.

When rubber products, including gaskets and seals, are exposed to an oily environment, particularly at high temperatures, destruction is associated with swelling of oil. The biochar-containing composites demonstrated a strong resistance to oil absorption by a reduction in equilibrium swelling after being immersed in motor oil at 90 °C for 4 days. In comparison to DP biochar, the results also demonstrated comparable results in the equilibrium swelling values for GR and RS biochar. The EPDM with 100% CB had around 107.9% swelling, whereas the lowest swelling values were 97–98%.

**Fig. 4:**
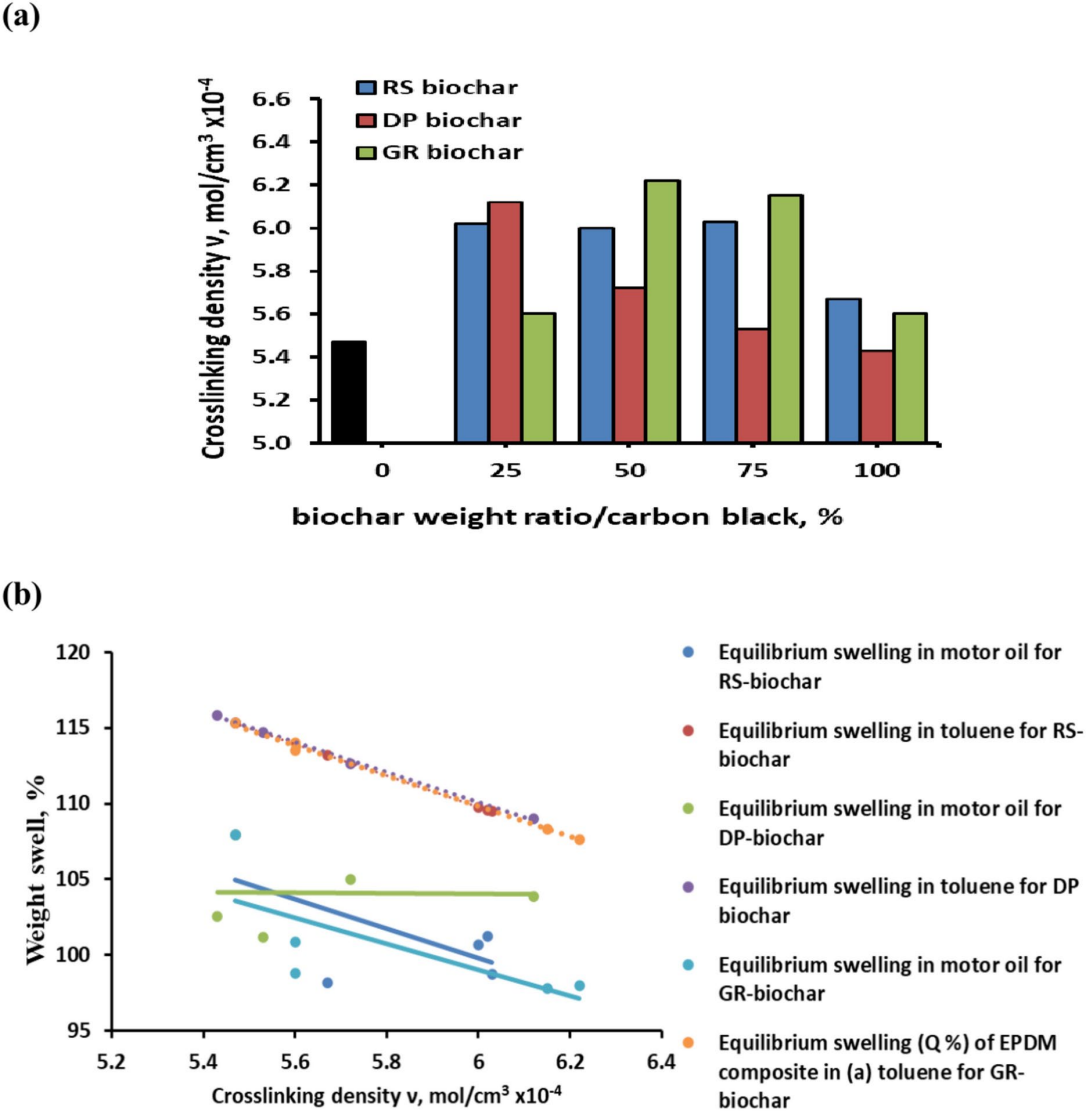
crosslinking density versus carbon black and biochar weight ratios.

Conversely, Fig. 4a shows the relationship between the weight ratio of biochar to carbon black and the crosslinking density. The graph shows that the crosslinking density of EPDM composites is positively impacted by biochar addition. This increase may be explained by the dispersion of the biochar throughout the matrix and making sufficient contact with the composites to enhance the interactions between filler and rubber and decrease the interactions between filler and filler^59^. For EPDM composites containing 25, 50, and 75% biochar as compared to 100% biochar, there is a noticeable and greater rise in the crosslinking density. This might be explained by the good distribution of the low ratios of biochar in the carbon black-polymer matrix, which causes the system to crosslinked well and strengthen the integrity of the crosslink structure. A relationship is illustrated between the swelling of the rubber composites and the crosslinking density. As shown in Fig. 4b, there was a negative linear relationship between the crosslink density and the swell ratios with high correlation coefficient, particularly with motor oil. This suggests that the low swelling of the rubber composite is caused by the high crosslink density between the rubber formulations.

#### Effect of biochar on thermo-oxidative ageing of EPDM composites

**Fig. 5:**
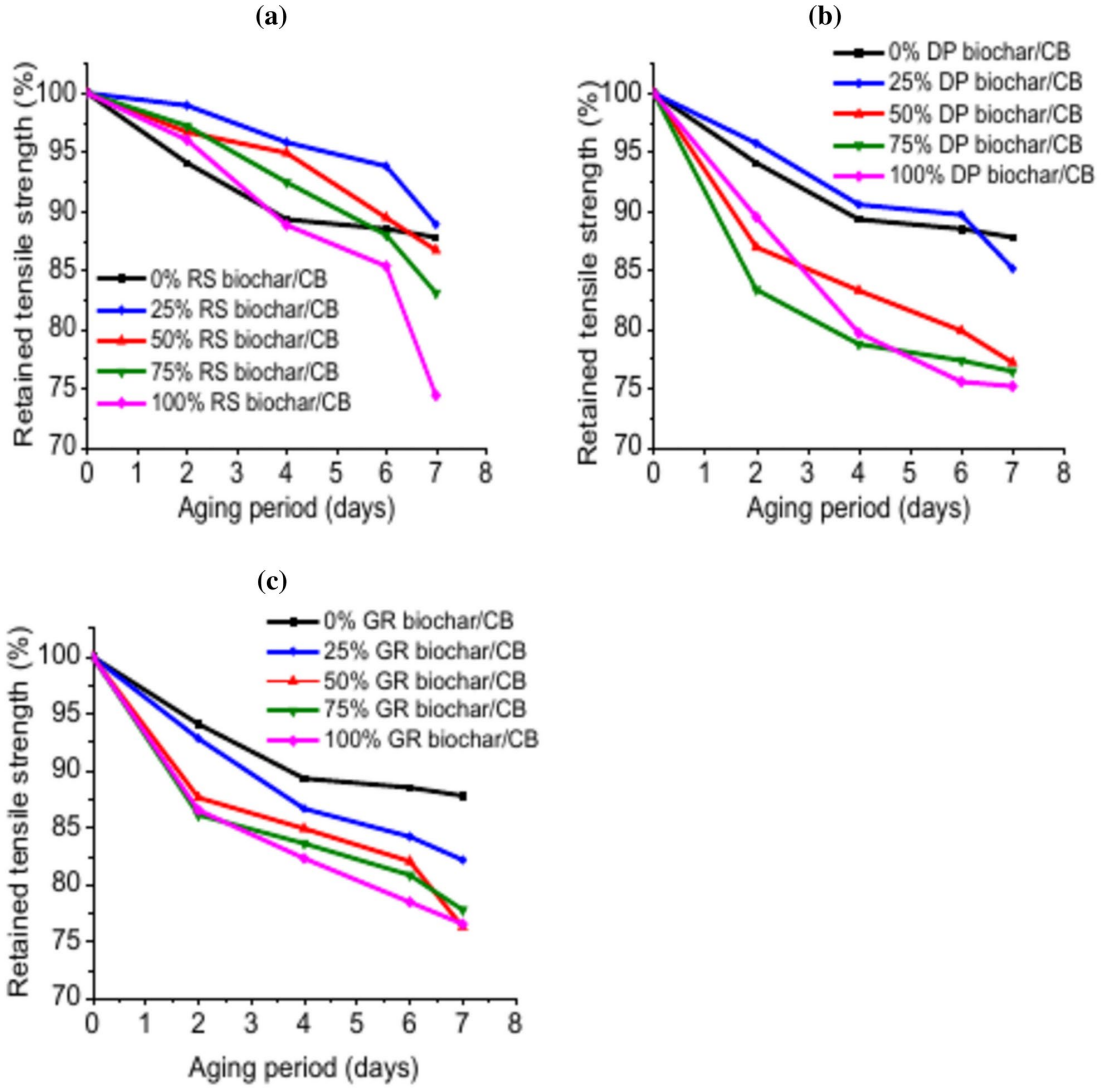
Retained values of tensile strength for EPDM filled with different biochar weight ratios.

**Fig. 6:**
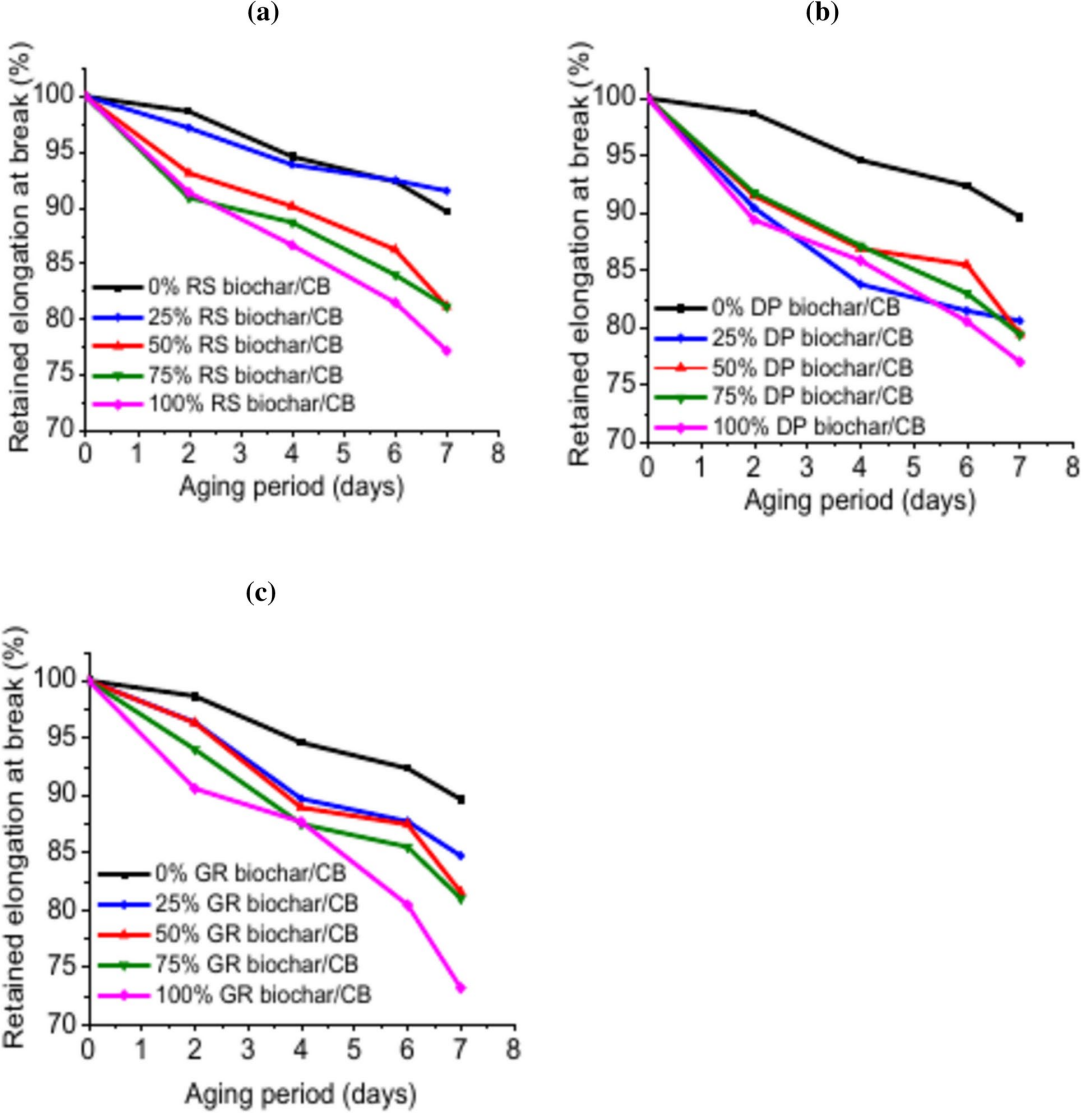
Retained values of elongation at break for EPDM filled with different biochar weight ratios.

One important test for rubber vulcanizates that has an impact on final product quality is thermal oxidative ageing. The retained values of tensile strength and elongation at break of the vulcanizates based on different weight ratios from biochar as reinforcing filler before and after aging are displayed in Figs. 5, 6, respectively. The vulcanizates rubber exhibits a relatively high degree of resistance to aging at 90 °C. Figure 5a–c shows that, after 7 days, EPDM containing (75CB/25 RS-biochar) had the greatest retained value of tensile strength (88.89%), followed by EPDM/(75CB/25 DP-biochar) (85.15%). Additionally, the figure reveals that EPDM vulcanizates with 25% RS and DP biochar exhibit better resistance to aging than EPDM vulcanizates with 100% carbon black, the conventional reinforcing agent.

Furthermore, the relationships between the retained values of elongation at break and various weight ratios from biochar are depicted in Fig. 6a–c. The result also demonstrated that the produced RS-biochar may enhance the ageing resistance of EPDM composites. The Figure shows that EPDM/(75CB/25RS) achieved the maximum retention value, followed by EPDM/100CB.

#### Tear strength (tear resistance)

**Fig. 7:**
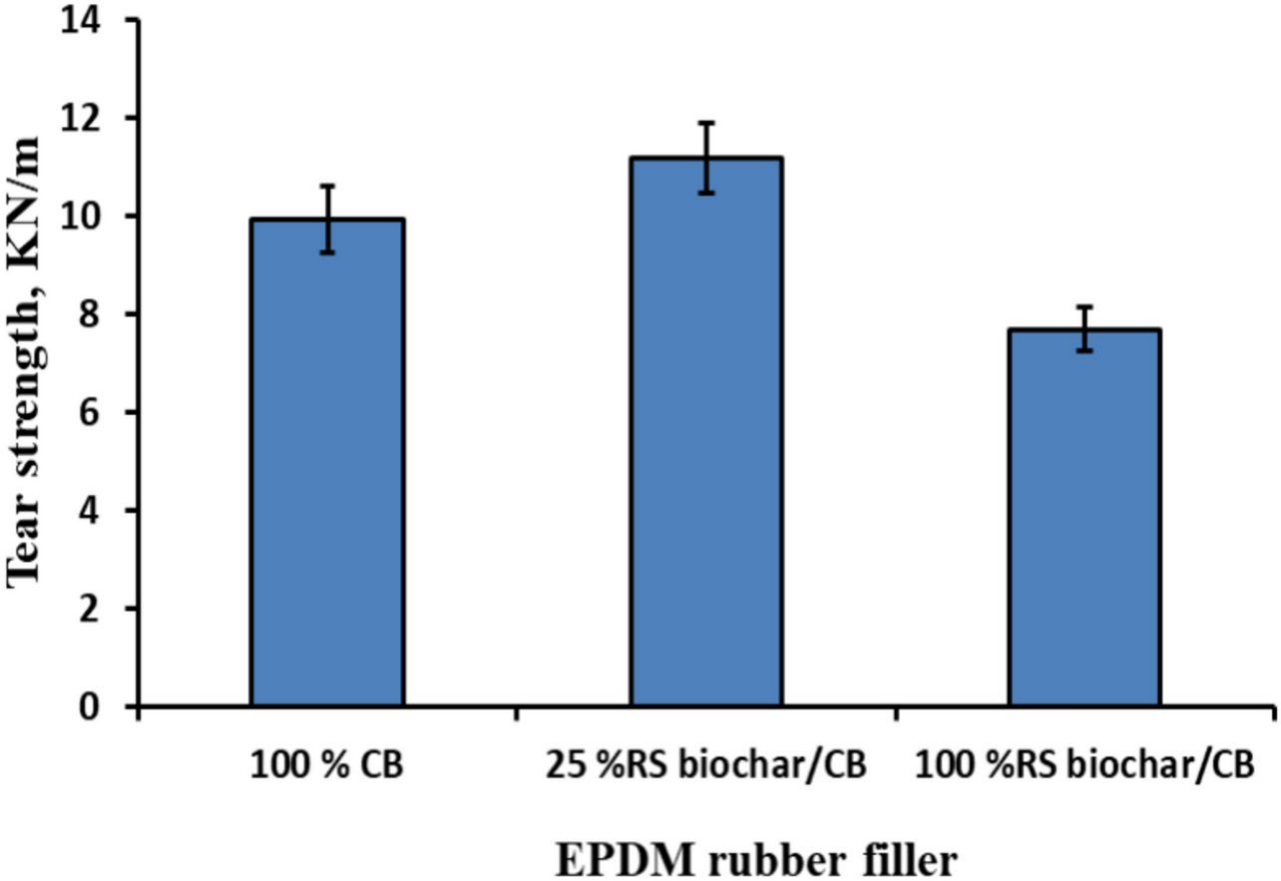
Tear strength of EPDM composites.

The maximal force needed to tear a test specimen in a direction normal to (perpendicular to) the direction of stress is known as rubber’s tear resistance. Figure 7 shows the tear strength of certain selected samples filled with 100% carbon black, 25% RS biochar, and 100% RS biochar. As anticipated, the trends for tear resistance and tensile strength are similar. The optimum sample with 25% biochar/CB had a tearing resistance of 11.19 KN/m, more than that of 100% CB and 100% RS (9.94 and 7.69 KN/m, respectively). As a result, the optimum condition sample gained a new value with tear strength results.

### Surface morphology analysis

**Fig. 8:**
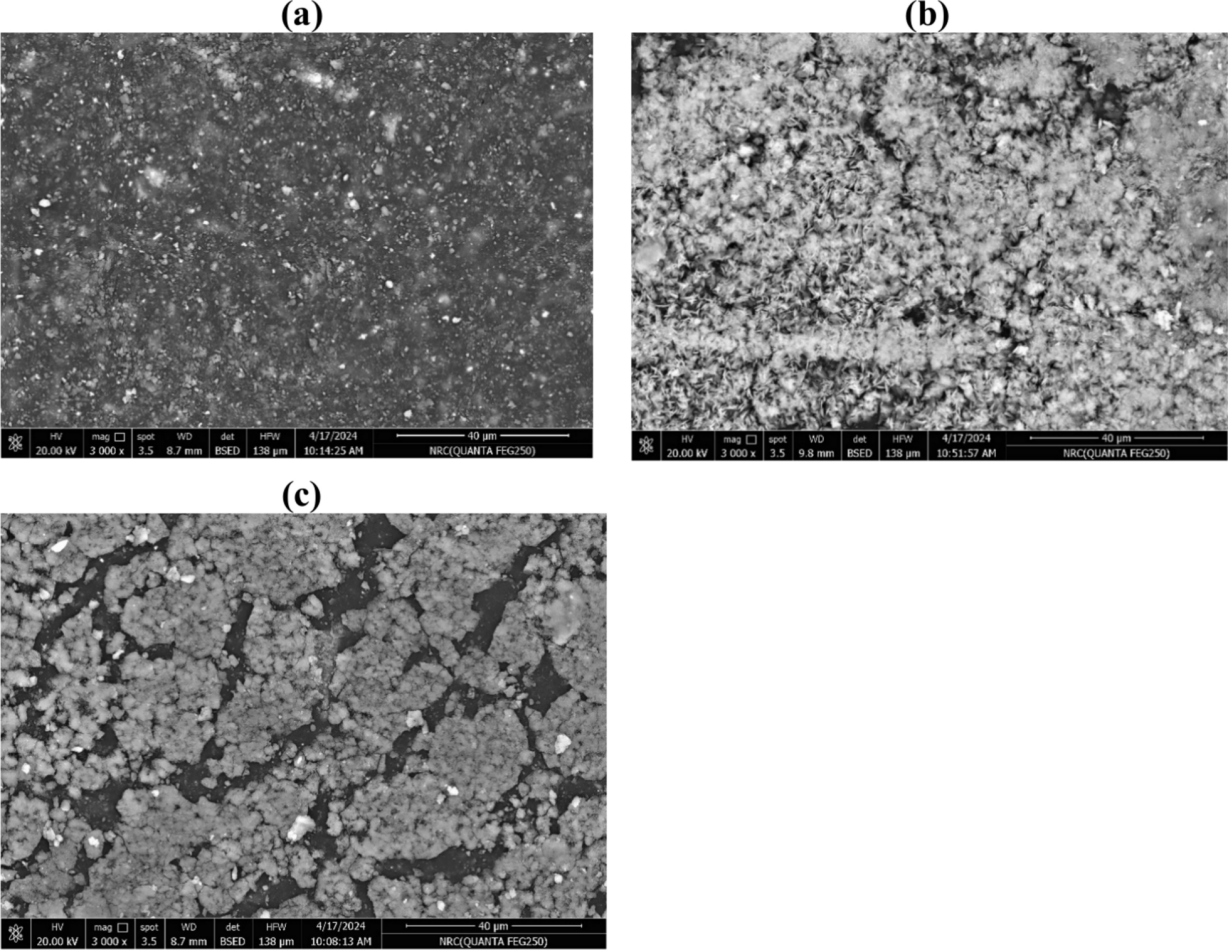
SEM of EPDM filled with (**a**) blank (100% carbon black) (**b**) 25% RS biochar (**c**) 100% RS biochar at magnification scale 40μm.

To demonstrate how the carbon black and biochar particles were distributed throughout the polymer matrix, SEM morphology was applied. The morphological and structural characteristics of the EPDM composite surface filled with 100% carbon black, 25% RS biochar and 100% RS biochar were assessed (Fig. 8). From the SEM images for the composite, the blank sample with 100% CB showed the matrix morphology is a more uniform surface of spherical microparticles of carbon provided reinforcing for the EPDM composite. For EPDM containing 25% RS-biochar, the biochar particles are evenly distributed with random orientation along the whole surface of the matrix. The surface showed a variety of crystals in needle shape that revealed the SiO_2_ content as illustrated by XRF analysis (22.4%). In composite incorporating 100% RS-biochar, a phase separated blend with non-continuous phase is observed due to the biochar aggregation. Therefore, due to the increased surface energy of 100% CB and 75CB-25 RS biochar particles as fillers, both of them appear to be distributed more evenly in the EPDM. While the presence of SiO_2_ content of the biochar can act as reinforcing filler.

## Conclusion

The utilization of sustainable filler resulting from agricultural wastes, namely rice straw, date palm fiber, and reed (*Arundo* *donax* L.), has the potential to decrease both the accumulation of these wastes in the environment as well as the health risk from using CB as filler. In this respect the agro-based biochar was used to serve as a co-filler in hybrid with CB allows the reduction of using non-renewable CB and improves the quality of existing rubber products. To achieve the optimal improvement in mechanical properties of EPDM rubber, a 25% RS-biochar substitution of CB is recommended; because beyond this content, the mechanical properties of rubber composites tend to deteriorate. It is interesting to note that the RS-based biochar showed promising results as a sustainable organic biofiller with improving the physical properties of EPDM rubber. This positive trend not noticed from literature on applying the biochars as filler in rubber purpose.

## Data Availability

All data generated or analyzed during this study are included in this published article.
